# Failed primary repair of blunt duodenal injury managed by tube duodenostomy, gastrojejunostomy and a feeding jejunostomy: a case report

**DOI:** 10.1186/s40792-024-01998-4

**Published:** 2024-08-23

**Authors:** Ngwane Ntongwetape, Elroy Patrick Weledji, Divine Martin Ngomba Mokake

**Affiliations:** Department of Surgery, Faculty of Health Sciences, University of Buea, S.W. Region, Buea, Cameroon

**Keywords:** Duodenum, Blunt injury, Repair, Fistula, Diversion, Tube duodenostomy, Gastrojejunostomy, Feeding jejunostomy

## Abstract

**Background:**

The worldwide increase in road traffic crashes and use of firearms has increased the incidence of duodenal injuries. Upper gastrointestinal radiological studies and computed tomography (CT) in resource settings may lead to the diagnosis of blunt duodenal injury. Exploratory laparotomy remains the ultimate diagnostic test if a high suspicion of duodenal injury continues in the face of absent or equivocal radiographic signs. Although the majority of duodenal injuries may be managed by simple repair, high-risk duodenal injuries are followed by a high incidence of suture line dehiscence and should be treated by duodenal diversion.

**Case presentation:**

We report a case of a failed primary repair of a blunt injury to the second part of the duodenum (D2) in a 24-year-old African man. This was successfully managed by a tube duodenostomy, a bypass gastrojejunostomy and a feeding jejunostomy in a low resource setting.

**Conclusions:**

Detailed knowledge of the available operative choices in duodenal injury and their correct application is important. When duodenal repair is needed, conservative repair techniques over complex reconstructions should be utilised. The technique of tube duodenostomy can be successfully applied to cases of large defects in the second part of the duodenum (D2), failed previous repair attempts and with defects caused by different aetiology. It may remain especially useful as a damage-control procedure in patients with multiple injuries, significant comorbidities and/or haemodynamic instability.

## Background

Complex duodenal injuries pose a significant challenge to the general surgeon and failure to manage it properly may have devastating results such as delayed leaks, widespread abdominal contamination, sepsis and death [1]. In addition, duodenal injuries are relatively rare and most surgeons would not have extensive experience in the management. Blunt injury of the duodenum is both less common and more difficult to diagnose than penetrating injury, and it may typically occur in isolation or with pancreatic injury. It usually occurs from crushing of the duodenum between spine and steering wheel, handle bar or some other force applied to the anterior aspect of the abdomen. Less commonly, deceleration may cause injuries (tears) at the junction of free (intraperitoneal) parts of the duodenum with fixed (retroperitoneal) parts (i.e. junction of the third and fourth part, and the first and second parts). A patient with a flexion/distraction fracture of L1–L2 vertebrae (the Chance fracture) or fractures of the transverse processes of the lumbar vertebrae are indicative of forceful retroperitoneal trauma and serve as a predictor of duodenal injury and should be closely observed [1, 2]. The total amount of fluid passing via the duodenum exceeds 6l/day and a fistula in this area may cause serious fluid and electrolyte imbalance. A large amount of activated enzymes liberated into the retroperitoneal space and the peritoneal cavity may be life-threatening. The upper portion of the duodenum that includes the first and second part has complex anatomical structures within it (common bile duct and sphincter) and the pylorus. It requires distinct manoeuvres to diagnose injury including quality contrast-enhanced computerised tomography (CT) imaging, cholangiogram, and, complex techniques to repair them. The first and second parts of the duodenum are densely adherent and dependent for their blood supply on the head of the pancreas, so diagnosis and management of any injury is complex, and resection, unless involving the entire C loop and pancreatic head, is impossible. The lower portion that includes the third and fourth part may generally be treated like small bowel. Diagnosis and management is relatively less complex, including debridement, closure, resection and re-anastomosis [2]. A preoperative diagnosis of isolated duodenal injury can be very difficult and there is no single method of duodenal repair that completely eliminates the possibility of dehiscence of the duodenal suture line. As a result the surgeon is confronted with the dilemma of choosing between several preoperative investigations and many surgical procedures. Detailed knowledge of the available operative choices and their correct application is therefore important [1, 2]. Intramural haematoma causing duodenal obstruction is a rare injury of the duodenum and specific to patients with blunt trauma [3]. In children, it is more common than duodenal perforation following trauma. This can be managed operatively by incising the serosa and expressing the haematoma or non-operatively with nasogastric aspiration and parenteral feeding until the haematoma resolves, usually over one to three weeks [4, 5]. Grading systems characterising duodenal injuries (Table 1) are less important clinically than the simple practical aspects of duodenal injuries: (a) the anatomical relation to the ampulla of Vater; (b) the characteristics of the injury (simple laceration versus destruction of duodenal wall); (c) the involved circumference of the duodenum; and (d) associated injury to the biliary tract, pancreas, or major vascular injury [1, 6]. Timing of the operation is also important as the mortality rate rises from 11–40% if the time interval between injury and operation is more than 24h [2, 7].

**Table 1:** Duodenal injury severity according to the American Association for the Surgery of Trauma[6]

Grade	Injury	Description
I	Haematoma Laceration	Single portion of duodenum Partial thickness only
II	Haematoma Laceration	Involving more than one portion Disruption < 50% circumference
III	Laceration	Disruption 50–75% circumference of D2 Disruption 50–100% circumference of D1, D3, D4
IV	Laceration	Disruption > 75% circumference of D2 Involving ampulla or distal common duct
V	Laceration	Massive disruption of duodenopancreatic complex Devascularisation of duodenum

D1, D2, D3, D4: first, second, third, and fourth portions of the duodenum. For multiple injuries, the grade is advanced by one

## Case presentation

**Fig. 1:**
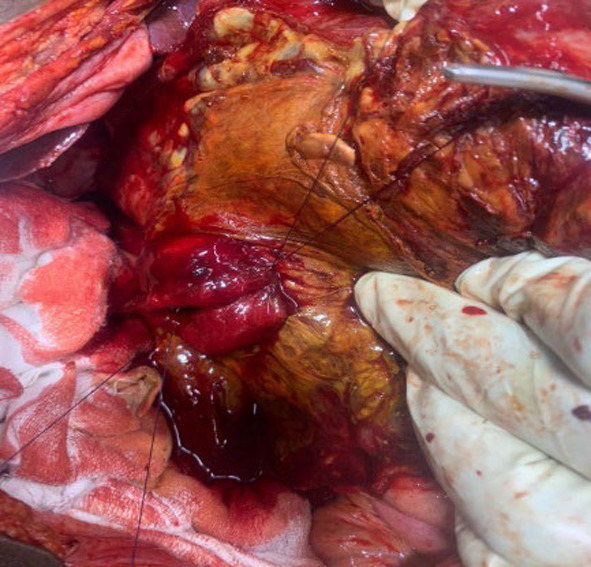
Second part of duodenum (D2) perforation with surrounding biliary soiling

**Fig. 2:**
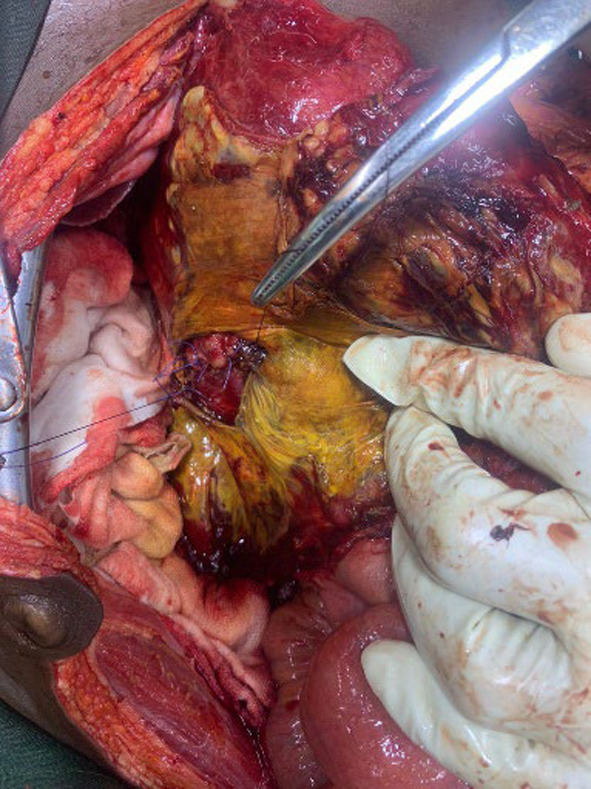
Primary repair of D2 perforation which failed

A fit 24-year-old African man was admitted as an emergency with worsening epigastric pain 48 h following a collision with the knee of a football opponent. He had no past medical history. On examination he was in distress with no mucocutaneous pallor. Apart from the vital signs revealing tachycardia (128/min), tachypnea (38 breaths/min), hypotension (100/70mmHg), pyrexia (38.5^0^C) and an oxygen saturation of 94%, chest and cardiovascular examination were normal. The abdomen moved with respiration but was diffusely distended with generalised tenderness but no rebound nor rigidity. Bowel sounds were present and digital rectal examination was unremarkable. Blood analysis revealed a haemoglobin of 12.2g/dl and a leukocytosis of 13.5 × 10^9^/l with neutrophilia. Clinical biochemistry including serum amylase were within normal range. A plain abdominal X-ray and an ultrasound of the abdomen were unremarkable. A computerised tomography (CT) scan was not available. A clinical diagnosis of peritonitis was made and following a rapid resuscitation he underwent a laparotomy 4 days following hospital admission. There was no free fluid, blood or pus in the peritoneal cavity but a dark-green discolouration with features of inflammation extending retroperitoneally from the posterior-lateral side of the caecum to the anterior abdominal wall and ascending towards the hepatic flexure of the colon. Following the mobilisation of the right colon a dark-greenish fluid gushed out into the abdominal cavity. The subsequent Kocherisation of the duodenum by dividing the lateral peritoneal attachment of the duodenum revealed a transverse perforation of ≈3cm in diameter involving ≈ 50–60% of the circumference of the second part of the duodenum (grade III disruption), distal to the ampulla of Vater (Fig. 1) with extravasating bilious content into the retroperitoneum (Table 1). Following debridement, the duodenal perforation was primarily repaired in two layers with interrupted 3.0 polygalactin (Fig. 2). A diverting gastrojejunostomy was performed and a nasojejunal tube inserted. Drains were placed adjacent to the repair. On postoperative day 4 he developed fever, tachycardia, and was tachypnoeic. Abdominal examination revealed a right flank tender swelling, dull on percussion and suggestive of an abdominal collection. A re-laparotomy showed a large bilious collection extending from the right paracolic gutter to the primary repair site at D2. Following a copious lavage a 16 Fr Foley catheter was inserted into the duodenal defect, secured with a purse-string 3.0 polygalactin suture and exteriorised via the right flank. A feeding jejunostomy tube was placed. The abdominal external drains were removed on day 7 as they became non-functional while the controlled duodenal fistula output gradually subsided. The tube duodenostomy was removed on day 21 post re-laparotomy after clamping for 2 days to ensure no adverse sequelae. He was discharged 20 days later following removal of the feeding jejunostomy tube.

## Discussion

A preoperative diagnosis of an isolated duodenal injury can be very difficult, and a high index of suspicion based on the mechanism of injury and physical examination may lead to further diagnostic studies. ‘Stomping’ and striking the mid-epigastrium is a common cause of blunt duodenal injury. The initial clinical changes in isolated duodenal injury may be extremely subtle before severe, life-threatening peritonitis develops as the duodenal contents extravasate into the peritoneal cavity as seen in this case [1, 8]. Non-operative management of an isolated low-grade duodenal injury is accepted with close clinical and laboratory monitoring of the patient [9]. Where the patient is unstable immediate laparotomy is required as the ultimate diagnostic test. Severe oedema, crepitation or bile staining of the periduodenal tissue implies a duodenal injury. Rapid staining of the periduodenal tissues following instillation of methylene blue via a nasogastric tube is evidence of intestinal leak in this area [10]. Nevertheless, mobilisation of the whole duodenum is mandatory initially with the Kocher manoeuvre [1, 11]. Entry into the lesser sac via the gastrocolic ligament provides exposure of the posterior aspect of the proximal portion of the first part of the duodenum and the medial aspect of the second part. Better inspection of the third and fourth parts of the duodenum entails mobilising the ligament of Treitz, the right colon including the hepatic flexure from right to left. This will enable the elevation of the right colon and small intestine and so expose the entire inframesocolic retroperitoneal organs, including the inferior vena cava (IVC), the right renal pedicle, the right iliac vessels, the duodenum, and the head of the pancreas (Cattell and Braasch manoeuvre) [12]. The small bowel mobilisation is undertaken by sharply incising its retroperitoneal attachment from the lower right quadrant to the ligament of Treitz. The majority of duodenal injuries may be managed by simple repair (closed transversely with one or two layers of slowly absorbing 3.0 polydioxanone (PDS) sutures). In complete transection of the duodenum, the preferred method of repair is primary anastomosis of the two ends after appropriate debridement and mobilisation. This is frequently with injuries of the first, third and fourth part of the duodenum where mobilisation is technically not difficult. If a large amount of tissue is lost, approximation of the duodenal ends may not be possible without undue tension on the suture line [1, 2, 9, 13]. Thus, complete transection in the first part of the duodenum may require an antrectomy with closure of the duodenal stump and a Billroth II gastrojejunostomy. When such injury occurs distal to the ampulla of Vater, a direct anastomosis to a Roux-en-Y loop sutured over the duodenal defect in an end-to-side fashion is the procedure of choice as mobilisation of D2 is limited by its shared blood supply with the head of the pancreas. This may also be applied as an alternative method to extensive defects of the other parts of the duodenum when primary anastomosis is not feasible [1, 2, 9]. External drainage with preferably a simple, soft silicone rubber, closed system placed adjacent to the repair should always be provided as it affords early detection and control of duodenal fistula [1, 2]. Although more complicated injuries require more sophisticated techniques, ‘damage control’ in high-risk duodenal injuries should precede more definitive reconstruction [1, 9]. High risk injuries are related to associated pancreatic injury, blunt or missile injury, involvement of more than 75% of the duodenal wall, time interval between injury and repair of > 24 h as in the index case, and associated common bile duct injury [1]. These are followed by a high incidence of suture line dehiscence and they should be treated by duodenal diversion [9].

**Fig. 3:**
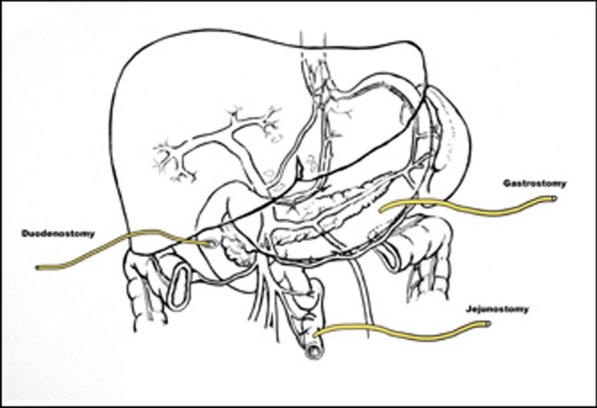
Schematic diagram illustrating ^‘^triple-ostomy^’^ in management of perforation in D2

A proximal tube decompression of the duodenum following primary repair is an old but underutilised technique known to decrease morbidity and mortality in patients with difficult to manage duodenal injuries. T-tube duodenostomy at site of perforation is also a simple technique that helps decompression following primary closure of duodenal perforation [14]. Duodenal diversion of the gastrointestinal contents with their proteolytic enzymes would protect the repair, and also make the management of a duodenal fistula easier [1, 2]. There are many other techniques described but most are complex procedures not appropriate for the management of an unstable patient who may require damage-control surgery. Tube decompression of the duodenum was initially utilised in management of the precarious closure of the duodenal stump after a gastrectomy, in order to prevent blow-out of the duodenal stump at the suture line or following a duodenal stump leak [15–20]. Over the years it has become safe and effective in the management of the difficult and complex duodenal injury which are generally more prone to leaks after repair than duodenal stumps after gastrectomy [16, 21]. In fact, a ‘triple-ostomy’ procedure which entails inserting a 14G Foley catheter in the duodenal perforation and bringing this out as a duodenostomy, a gastrostomy using a 14 G Foley catheter (removing the need for long-term use of a nasogastric tube) and a 9Fr jejunostomy tube placed for post- operative feeding is a recommended alternative for the emergency repair of the rare D2 peptic ulcer perforation which is also associated with life-threatening complications after conventional surgical operations (Fig. 3) [21–25]. Significant loss of the second part of the duodenum ( D2) may require different techniques [1, 25], the simplest of which is an on-lay patch of the jejunum. The serosal surface of the jejunum is anastomosed directly to the defect in the duodenal wall. There is considerable evidence of leakage with this technique [26] and, where feasible, a Roux- en-Y loop drainage of the defect is procedure of choice in the stable patient [27]. If there is concern about the possibility of leakage, e.g. tension on the suture line, a tube duodenostomy can be used to create a controlled duodeno-cutaneous fistula as in this case [21, 24]. An alternative to this is pyloric exclusion by internal closure of the pylorus effected through a gastrostomy. The pyloric ring is closed with a continuous suture or stapling which breaks down after several weeks regardless of the material used [28]. Most authors combine it with a gastrojejunostomy [29] and few cover this period with a nasogastric tube. Recent studies have found no difference in mortality with primary repair and pyloric exclusion compared to just primary repair [30]. The addition of octreotide and intravenous acid suppression may improve duodenal healing and decrease stomach ulceration. Both pancreaticoduodenectomy (PD) and pancreas preserving duodenectomy (with reduction in the number of anastomoses and avoidance of manipulation of the biliary tree) require extensive knowledge of the anatomy and familiarity with operations in this region and are not feasible options in the haemodynamically unstable patient [31–33]. They should be considered only if unavoidable (grades IV and V) and, in fact, much of the dissection must have been done by the wounding force [1, 11]. These include massive disruption of the pancreaticoduodenal complex, devascularisation of the duodenum and, sometimes, extensive injury to the second part of the duodenum involving the ampulla or distal common bile duct [32, 33]. Extensive local damage of the intraduodenal or intrapancreatic bile duct frequently necessitates a staged PD [31]. Less extensive local injuries may be managed by intraluminal stenting, sphincteroplasty or the re-implantation of the ampulla of Vater [1]. A few patients with grades IV and V are eventually salvaged by drainage and parenteral nutrition and meticulous overall care than by a desperate PD [1, 10, 34]

The Roux-en-Y duodenojejunostomy, initially defined in 1975 [27] has been regarded as a safe alternative and the operation of choice with penetrating D2 injuries, but there is no data regarding its use in unfavourable conditions such as delayed diagnosis, giant ulcers, re-leak where significant inflammation is present and in disseminated tumour cases where the tissue healing is grossly impaired [35]. In addition, the technique adds one more anastomosis with a risk of leak in the patient already suffering a hostile abdomen.

The underutility of tube duodenostomy is due to the successful use of duodenojejunostomy for the management of such defects in both trauma and tumour invasion and in very large defects not amenable to tube duodenostomy. The early literature on tube duodenostomy for D2 injuries also showed no change in outcome and with the high leak rates likely contributed to the lack of popularity. However, an omental flap placed around the exit site of the tube in the duodenum or the Witzel approach would prevent leakage around the site and further secure the drain in place [21]. In addition, the duodenal drain should be left in place for a minimum of 6 weeks in order for a defined track to develop, similar to the use of T tubes in bile duct injuries and successful treatment confirmed by no leakage after tube clamping. A tube duodenostomy via a perforation of > 3cm is a simple technique that does not involve an anastomosis and the addition of diversion of gastric contents by either pyloric exclusion, tube gastrostomy or a gastrojejunostomy as in this case may improve its efficacy [21–24]. In addition, a feeding jejunostomy is useful in view of the time (4–6 weeks) expected for duodenal healing. The fashioning of a feeding jejunostomy at the initial laparotomy in patients with duodenal injury and extensive abdominal trauma (abdominal trauma Index greater than 25 is highly recommended [1, 22–25]. Tube duodenostomy may provide an opportunity to stabilise the patient, converting an impending catastrophe to a future elective surgery where the possibility of transfer to a sub-specialty expertise exists [21, 22]. It also provides a safe alternative to complex surgery in low resource centres as in the index case.

## Conclusions

The technique of tube duodenostomy can be successfully applied to cases of large defects in the second part of the duodenum (D2), failed previous repair attempts and with defects caused by different aetiology, including blunt trauma, peptic ulcer disease and erosion from cancer without concomitant pyloric exclusion. Despite good outcome it has been underutilised. It may, however, remain especially useful as a damage-control procedure in patients with multiple injuries, significant comorbidities and/or haemodynamic instability.

## Data Availability

Data will be made available by the corresponding author upon request.

## Abbreviations

CT: Computed tomography
D1: First part of duodenum
D2: Second part of duodenum
D3: Third part of duodenum
D4: Fourth part of duodenum
PD: Pancreaticoduodenectomy
IVC: Inferior vena cava
PDS: Polydioxanone suture
